# Supplementary material for: COVID-19 Alcoholic Cirrhosis and Non-Alcoholic Steatohepatitis Cirrhosis Outcomes among Hospitalized Patients in the United States: Insight from National Inpatient Sample Database
Source: Trop Med Infect Dis. 2022 Dec 7;7(12):421. doi: 10.3390/tropicalmed7120421 (PMC9786037; doi:10.3390/tropicalmed7120421)
Supplement: Supplementary file 1 [file tropicalmed-07-00421-s001.zip › tropicalmed-2063089-supplementary.pdf]

Tropical Medicine and Infectious Disease

MDPI

Supplementary Materials for COVID-19 Alcoholic Cirrhosis and Non-Alcoholic Steatohepatitis Cirrhosis Outcomes among Hospitalized Patients in the United States: Insight from National Inpatient Sample Database

Table S1. ICD 10 codes.

Variable	ICD-10 CM Code
COVID	U071, U00, U49, U50, U85, J1282
Alcoholic liver cirrhosis	K70.3XX
NASH	K74.6XX, K75.81
Hyponatremia	E87.1
Portal HTN	K76.6
Hepatorenal syndrome	K76.7
Portal vein thrombosis	I81
SBP	K65.2
Variceal bleed	I85.01, I85.11
Shock	R57.XX, R65.21
Hepatic encephalopathy	K72.01, K72.91
Smoking	F17.XX, Z87.891
CAD	I25.10, I25.11, I25.118, I25.119, I252, I253, I25.4XX, I25.5, I25.6, I25.8XX, I25.7XX, I25.9XX
CHF, HTN, DM, CKD, Chronic pulmonary disease, Obesity	Elixhauser comorbidities were used
AKI	N17.XX, N99.0
**Variable**	**ICD-10 procedure code**
Intubation	5A1945Z, 5A1955Z, 5A1935Z, 5A09357, 5A09457, 5A09557
Vasopressor use	3E030XZ, 3E033XZ, 3E040XZ, 3E043XZ, 3E050XZ, 3E053XZ, 3E060XZ, 3E063XZ
Hemodialysis	5A1D70Z, 5A1D90Z, 5A1D80Z, 5A1D00Z, 5A1D60Z

ICD codes used for different diagnosis.
